# Adding Mobile Elements to Online Physical Activity Interventions for Adults Aged Over 50 Years: Prototype Development Study

**DOI:** 10.2196/42394

**Published:** 2023-01-25

**Authors:** Eline H G M Collombon, Denise A Peels, Catherine A W Bolman, Gert-Jan de Bruijn, Lilian Lechner

**Affiliations:** 1 Faculty of Psychology Open Universiteit Heerlen Netherlands; 2 Department of Communication Science University of Antwerp Antwerp Belgium

**Keywords:** prototype, pilot test, eHealth, mHealth, physical activity, older adults, development, usability

## Abstract

**Background:**

Only a minority of adults aged over 50 years meet physical activity (PA) guidelines of the World Health Organization (WHO). eHealth interventions are proven effective tools to help this population increase its PA levels in the short term, among which the Active Plus and I Move interventions have been developed by our own research group. To achieve long-term effects, increase intervention use, and decrease dropout rates, 3 emergent but different mobile elements (an activity tracker, an ecological momentary intervention [EMI] program, and a chatbot) were added separately to Active Plus and I Move. In this study, the prototype development and pilot-testing of these interventions is described.

**Objective:**

This study aims to enhance 2 existing PA-stimulating computer-based interventions with 3 mobile elements (an activity tracker, an EMI program, or a chatbot) and test the prototypes on usability and appreciation within a target population of adults aged over 50 years.

**Methods:**

A systematic design protocol consisting of development, evaluation, and adaptation procedures was followed with involvement of the target population. Literature searches separated per mobile element and interviews with the target population (N=11) led to 6 prototypes: Active Plus or I Move including (1) an activity tracker, (2) EMI, or (3) a chatbot. These prototypes were tested on usability and appreciation during pilot tests (N=47) and subsequently fine-tuned based on the results.

**Results:**

The literature searches and interviews provided important recommendations on the preferences of the target population, which enabled us to develop prototypes. The subsequent pilot tests showed that the mobile elements scored moderate to good on usability, with average System Usability Scale (SUS) scores of 52.2-82.2, and moderate to good on enjoyment and satisfaction, with average scores ranging from 5.1 to 8.1 on a scale of 1-10. The activity tracker received the best scores, followed by EMI, followed by the chatbot. Based on the findings, the activity tracker interventions were fine-tuned and technical difficulties regarding EMI and the chatbot were solved, which is expected to further improve usability and appreciation.

**Conclusions:**

During this study, 6 prototypes of online PA interventions with added mobile elements were developed and tested for usability and appreciation. Although all prototypes scored moderate to high on usability, enjoyment, and satisfaction, it can be concluded that the integration of an activity tracker with a computer-based PA intervention is the most promising option among the 3 mobile elements tested during this study. The prototype development steps of the systematic design protocol followed can be considered useful and successful for the purposes of this study. The interventions can now be evaluated on a larger scale through a randomized controlled trial.

**International Registered Report Identifier (IRRID):**

RR2-10.2196/31677

## Introduction

Physical inactivity has been shown to be a major predictor for mortality in adults aged over 50 years [1]. According to the World Health Organization (WHO) guidelines, they should be physically active from moderate to vigorous levels for at least 150 minutes per week. Additionally, they are recommended to train for strength and flexibility at least 2 times per week [2]. However, it has been shown that only a minority of adults aged over 50 years meet these guidelines [3]. Increasing physical activity (PA) levels among this population is thus of major importance and is associated with improved physical, functional, psychological, and cognitive health [4-6].

eHealth interventions are proven effective tools to help adults aged over 50 years increase their PA levels in the short term, being the first weeks after completion of the intervention [7]. Additionally, these online interventions have potential advantages compared to traditional interventions, including a wide reach, more accessible information, and reduced workload for practitioners [8,9]. In the past decade, our research group has also developed online interventions to promote PA levels focused on different target populations [10-13]. Although the interventions were proven effective in increasing PA levels in the short term, these positive effects were not maintained in the longer term [10,11]. Therefore, during this study, the online interventions *Active Plus* [14] and *I Move* [15] were renewed by separately adding 1 of 3 mobile elements to the web-based interventions. As a result, it is expected that (long-term) effects on PA improve, intervention use increases, and attrition rates decrease. Additionally, this is in line with the recent increases in smartphone and tablet ownership observed in adults aged over 50 years [16].

*Active Plus* is a computer-based intervention targeted at adults aged 50 years and older and includes the provision of tailored advice regarding PA on 3 occasions delivered during 3 months. Tailoring takes place based on the answers of users on questions regarding, for example, demographics, diseases and limitations, current PA levels, the intention to be (more) physically active, and preferences for exercise activities [14]. *I Move* is a more interactive computer-based intervention targeted at adults, based on motivational interviewing and self-determination theory [17,18]. *I Move* comprises 4 sessions regarding PA, delivered during 3 months, where users are given information about various topics, such as planning, current PA behavior, barriers, and videos with narratives on confidence and motivation related to PA [15]. More information about *Active Plus* and *I Move* can be found in Multimedia Appendices 1 and 2.

One of the selected mobile elements to integrate with *Active Plus* and *I Move* is an activity tracker with an accompanying app. In addition to the proven effectiveness in increasing PA [19,20], it has been shown that older adults are willing to use this tool [21-23]. An ecological momentary intervention (EMI) program was developed and integrated with *Active Plus* and *I Move* as a second mobile element. Although EMI is often used and researched to help with smoking cessation [24,25] or to reduce alcohol consumption [26], less is known regarding its effectiveness in improving PA behavior. Lastly, a chatbot app that delivers persuasive tailored walking messages throughout the day was integrated with *Active Plus* and *I Move*. These mobile interactive virtual coach apps are promising tools with regard to the stimulation of PA [27].

**Figure 1:**
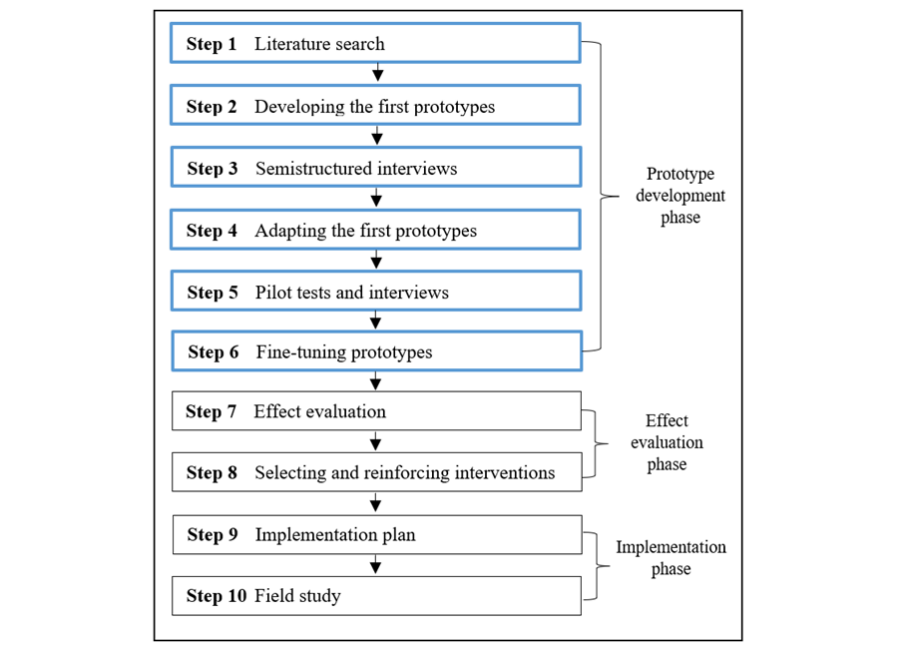
Schematic overview systematic design approach with the prototype development phase highlighted.

As described in a design paper [28], during the renewal of the interventions, a systematic design approach was used to preserve and further strengthen the already proven effectiveness of the computer-based *Active Plus* [10] and *I Move* [11] interventions. Efforts were made to involve the target population, which has been shown to be an essential factor for successful eHealth and mobile health (mHealth) development [29,30]. Roughly, the applied systematic approach was divided into a 6-step prototype development phase, a 2-step effect evaluation phase, and a 2-step implementation phase, which is shown in Figure 1.

In this paper, the prototype development and pilot-testing of the interventions is described. The aim was to enhance 2 existing PA-stimulating computer-based interventions with 3 mobile elements (an activity tracker, an EMI program, or a chatbot) and test the prototypes on usability and appreciation within the target population of adults aged over 50 years.

## Methods

The prototype development phase of the systematic design approach [28] comprised 6 steps. A schematic overview of these steps and their relationship to subsequent steps of the systematic approach is provided in Figure 1. Methodological procedures per step are described next.

### Steps 1 and 2: Literature Search and Development of the First Prototypes

Literature searches regarding the additional mobile elements were performed to provide a base for development of the first prototypes. For the activity tracker, a market study on available trackers in the Netherlands with a maximum price of approximately 100 euros (US $106.41) each was performed (unpublished). Results of the market study were applied on a literature search, where particular attention was paid to design preferences, perceived easiness of use, and attitude toward activity trackers within the population of adults aged over 50 years. Based on this literature search and market study, an appropriate activity tracker was selected to be integrated with *Active Plus* and *I Move*. Costs of the activity trackers in relation to usability were also considered during this phase. The development of the EMI prototype was based on a literature search where the barriers and motivators for adults aged over 50 years to participate in PA were studied. Additionally, the existing literature regarding EMI use for PA promotion and the use among adults aged over 50 years was studied. For the chatbot, an already existing app originally developed for the Supreme Nudge project [31] was adapted according to the study needs and integrated with the online interventions. Since the chatbot was developed according to a systematic design process, the literature search phase for this element had already taken place during its original design process [27]. As a result, more attention was paid to step 2 of our systematic approach, where the element was adapted to fit the study demands. Additionally, emphasis was put on achieving a high degree of integration of the chatbot with our existing online interventions.

### Steps 3 and 4: Semistructured Interviews and Adapting the First Prototypes

Semistructured interviews regarding the 3 mobile elements were held among the target population of adults aged 50 years and older. The aim of these interviews was to improve the first prototypes and better meet the preferences of the target population. Recruitment took place via online advertisements, and the sample size was based on when thematic saturation was reached [32]. We tried to include a sample of participants who varied by characteristics, such as level of education, age, gender, and digital skills (Table 1). Prior to the interview, participants signed an informed consent form and filled in a questionnaire consisting of questions related to sociodemographic variables and digital skills, based on a combination of validated tools, such as the Digital Health Literacy Instrument [33]. A semistructured interview guide based on models, such as the unified theory of acceptance and use of technology (UTAUT) [34] and the technology acceptance model (TAM) [35] was followed for the first mobile element (ie, the activity tracker; see Multimedia Appendices 3 and 4). During the interview, the first prototypes and accompanying manuals were tested by interviewees and their usability was discussed. Based on the input of the interviewees and the time needed to discuss the activity tracker, it was decided whether the EMI or chatbot element could be discussed as well during the 45-60-minute range of 1 interview. In case not all mobile elements could be covered within the time frame of 1 interview, participants were asked whether they gave permission for a follow-up interview. All concerning participants (N=4) gave permission for this second interview (Table 1). The interviews were audio-taped and transcribed verbatim, where names and other personal information about the participants were pseudonomized. First, the transcripts were read for familiarization. Subsequently, an initial thematic framework was developed based on the semistructured discussion guide and the familiarization procedure. Afterward, the transcripts were coded by one researcher and checked by another researcher, and any uncertainties or inconsistencies were discussed. The resulting data were used to improve the first prototypes of the renewed versions of *Active Plus* and *I Move*.

**Table 1:** Characteristics of participants.

Characteristics		Participants (N=11)
**Sociodemographic variables**		
	Gender (men), n (%)	8 (73)
	Level of education (low educated), n (%)	2 (18)
	Age (years), means (SD)	59.1 (3.4)
	1 interview needed to discuss 3 mobile elements, n (%)	7 (64)
	2 interviews needed to discuss 3 mobile elements, n (%)	4 (36)
**Digital skills questionnaire^a^ score, mean (SD)**		
	Handling a smartphone (1=really poor to 10=perfect)	6.8 (1.1)
	Use of the internet (1=really poor to 10=perfect)	7.3 (1.2)
	Installation and use of apps (1=very difficult to 10=very easy)	7.0 (1.4)
	Frequency of using health apps (1=never to 10=very often)	3.6 (2.3)
	Searching health information on the internet (1=very difficult to 10=very easy)	7.5 (1.1)

^a^Based on a combination of validated tools (eg, Digital Health Literacy Instrument).

### Step 5: Pilot Tests and Interviews

The 6 renewed interventions (*Active Plus*+activity tracker, *Active Plus*+EMI, *Active Plus*+chatbot; *I Move*+activity tracker, *I Move*+EMI, and *I Move*+chatbot) were pilot-tested for usability and acceptability by the target population of adults aged over 50 years. Previous interview participants of step 3 who gave consent to approach them again were invited to participate in step 5, supplemented with recruitment via social media advertisements. We aimed for a sample with a variation in factors, such as age, gender, and level of education. An overview of the characteristics of the pilot test participants is shown in Table 2. Although some variation was found between groups regarding baseline minutes of the moderate-to-vigorous physical activity (MVPA) per week, 1-way ANOVA showed no significant differences (*P*=.83).

**Figure 2:**
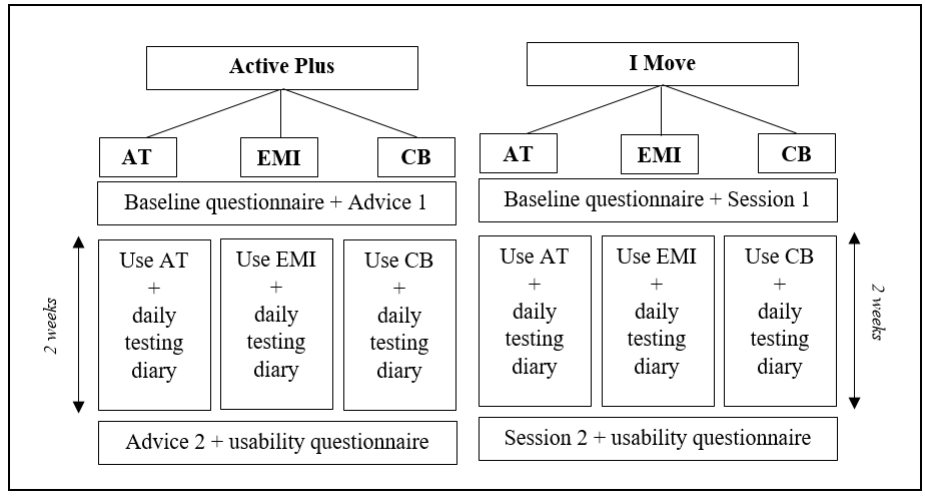
Schematic overview of the procedure of pilot tests. AT: activity tracker; CB: chatbot; EMI: ecological momentary intervention.

For the pilot tests, shortened interventions with a duration of 2 weeks were used instead of the original 12 weeks (Multimedia Appendices 1 and 2). As a result, participants in the *Active Plus* interventions only received the first and the second advice and participants in the *I Move* interventions only followed the first and the second session. In the intervening 2 weeks, participants used 1 of the mobile elements: the activity tracker or EMI or the chatbot. Prior to the first advice or session, participants filled in an online baseline questionnaire (T_0_) assessing, among other things, demographic variables and PA levels via the short questionnaire to assess health-enhancing physical activity (SQUASH) [36]. During the 2-week use period of the assigned mobile element, participants were instructed to fill in a short daily paper-based testing diary consisting of questions related to experiences, technical issues, and usability of the mobile element on that specific day. They received this testing diary via post at the start of the test, together with instruction manuals for the mobile element. Directly after receiving the second advice of *Active Plus* or completing the second session of *I Move*, participants were asked to fill in an online usability and acceptability questionnaire. This questionnaire and the daily testing diary were both based on a combination of validated tools and models, such as the System Usability Scale (SUS) [37], the TAM [35], and the UTAUT [34], earlier extensively described in a design protocol paper [28]. A schematic overview of the pilot tests procedure is shown in Figure 2.

**Table 2:** Characteristics of participants in the pilot tests.

Characteristics		Activity tracker (n=18)	EMI^a^ (n=15)	Chatbot (n=14)
Gender (male), n (%)		7 (39)	4 (27)	3 (21)
Age (years), mean (SD)		62.3 (7.4)	56.1 (4.4)	58.5 (7.0)
BMI (kg/m^2^), mean (SD)		28.3 (4.1)	29.1 (5.7)	27.6 (3.4)
**Level of education**				
	Low	2 (12)	1 (7)	1 (7)
	Middle	8 (44)	5 (33)	3 (21)
	High	8 (44)	9 (60)	10 (72)
MVPA^b^ T_0_^c^ (minutes per week), mean (SD)		925.2 (452.1)	945.9 (630.1)	720.9 (417.1)
Intention to be more physically active T_0_ (1-10), mean (SD)		8.2 (1.7)	8.3 (0.9)	8.4 (1.0)

^a^EMI: ecological momentary intervention.

^b^MVPA: moderate-to-vigorous physical activity.

^c^T_0_: baseline questionnaire.

A sample of pilot-test participants who gave consent for approaching them again after completing the pilot test were invited to participate in a semistructured interview that was also part of step 5. The aim of this interview was to obtain more in-depth information about the usability and acceptability of the mobile elements and renewed interventions, in addition to the already completed questionnaires during the pilot test. The sample size was based on when thematic saturation was reached (N=3 per mobile element). A semistructured discussion guide was followed, and the same procedures regarding audio-recording and data analysis were applied as during the previous semistructured interviews of step 3.

### Ethical Considerations

All aforementioned procedures, being part of the prototype development phase of the systematic design approach [28], were approved by the central ethical review committee of the Open University of the Netherlands (approval no. U202004903, approval date July 7, 2020). Additionally, all data were obtained and stored anonymously according to the composed data management plan and following the General Data Protection Regulation (GDPR). All participants provided informed consent for participation in the study. Interview participants received a small gift in the form of a drinking bottle. Among the pilot-test participants, 3 book vouchers worth 15 euros (US $15.96) were raffled.

## Results

### Intervention Including the Activity Tracker

In the subsequent sections, the results of the prototype development phase of the intervention including the activity tracker are described.

#### Steps 1 and 2: Literature Search and Development of the First Prototypes

First, a market study on available trackers in the Netherlands was performed (January 2020). Considering future implementation of our interventions in practice (eg, municipalities) combined with our focus on groups with low socioeconomic status, a selection of activity trackers was made based on price as well. Only trackers with a maximum price of approximately 100 euros (US $106.41) were included in the preselection shown in Multimedia Appendix 5. The subsequent literature search was based on the preselected trackers, which is summarized in this section and in Multimedia Appendix 5.

Considering our target population of adults aged 50 years and over with often lower digital skills and the aim to improve PA behavior, we decided to focus the activity tracker intervention element only on the step count and not on other parameters, such as calorie estimates and heart rate. The aim of focusing on only the step count was to prevent overloading participants with information. As a result, particularly the step count of the preselected trackers was studied and no other parameters. Additionally, it has been shown that the step count is the most popular parameter among activity trackers [38]. However, this parameter is associated with some disadvantages as well, which mainly concern the elderly: measurements can be influenced due to altered walking patterns [39,40], and it could be difficult to measure the step count validly during low walking speeds (<0.8 m/s) [41]. Despite these possible drawbacks, it appears that the most frequently investigated Fitbit devices are highly valid for measuring steps in older adults [42]. The mean absolute percentage error for step counting of our preselected Xiaomi devices is comparable to that of a Fitbit device [43], and the accuracy is good in proportion to the low price [44]. Although studies on the accuracy of measuring the step count with Fitbit and Xiaomi devices were found during the literature search [42-44], substantially fewer studies were found regarding accuracy of the preselected Huawei, Samsung, and Garmin trackers for the target population of older adults in our literature search performed in January 2020.

Considering the decline of vision with age, display type, screen size, and resolution are crucial factors for the usability of the tracker for our target population. Multimedia Appendix 5 shows that Xiaomi Mi Smart Band 4 and Huawei Band 3 Pro are both featured with a colored screen, high screen resolution, and big screen size. Additionally, costs of the tracker are important due to future large-scale implementation of our renewed interventions in practice. As a result, the earlier mentioned advantages of Fitbit Inspire, Huawei Band 3 Pro, Fitbit Charge 3, and Garmin Vivosport do not outweigh the costs compared to the less expensive devices. Xiaomi Mi Smart Band 4 with the accompanying Mi Fit app scores the best among the remaining less expensive trackers, since accuracy is improved [43] and screen size and resolution are sufficient. Combined with a battery life of 20 days and the little time needed to charge, we decided to integrate Xiaomi Mi Smart Band 4 with *Active Plus* and *I Move*. Based on this choice, a manual (manual A) for installation and use of the tracker and the accompanying Mi Fit app was developed. For these step-by-step instructions, short sentences [45], a large font, and a combination of textual and visual elements were used in order to reach both those with low literacy and those with high literacy [46]. Additionally, helpdesk contact details were provided in case the users encountered difficulties or had other questions.

Most trackers are equipped with the behavior change techniques (BCTs) of goal setting, feedback, rewards, self-monitoring, and social support, which are all considered valuable for reinforcing the original *Active Plus* and *I Move* interventions [47]. Within the Mi Fit app, users can connect with other Mi Fit users, which is expected to increase social support. With regard to goal setting, it is important that users can adapt the step goal to fit their personal situation instead of having an automatically determined goal within the software. Therefore, 2 paper-based manuals were developed to guide users in setting feasible step goals and help with self-monitoring. In manual B, instructions are based on intensive use of the Mi Fit app, where participants are instructed to extract weekly average steps from the app. Participants less familiar with the use of apps can use manual C, where day-to-day paper-based schemes are provided and weekly average steps need to be calculated manually. With regard to self-monitoring and rewards, advisory texts were developed for the advices of *Active Plus* and the sessions of *I Move*, in which users receive feedback on whether their number of steps has increased or decreased in time and whether a further increase in their steps could benefit their health. In addition to textual advice, visual elements were included as well. The advisory texts are based on a number of questions related to the activity tracker and the measured steps, which were added to the intervention questionnaires. Examples of the advisory step count texts and visuals within the interventions are provided in Multimedia Appendix 6.

#### Steps 3 and 4: Semistructured Interviews and Adapting the First Prototypes

**Figure 3:**
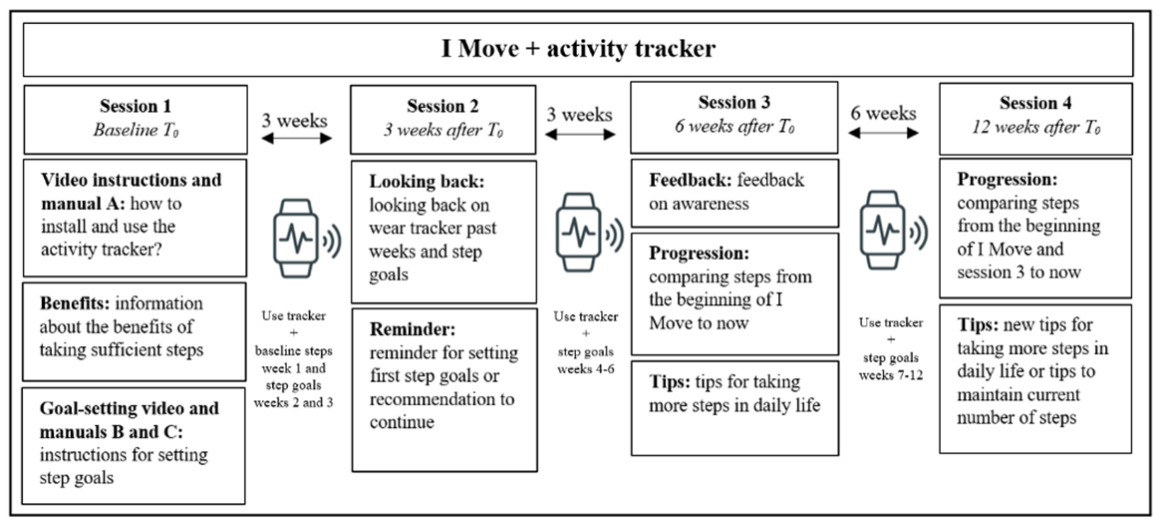
Overview of the online intervention I Move including the activity tracker.

The semistructured interviews resulted in numerous suggestions and findings regarding the interventions including the activity tracker, resulting in the following themes: (1) user experiences, (2) attitude and preferences, (3) application, (4) opinion on the testing procedure, (5) instruction manual, (6) reaching the target population, (7) step goals, and (8) combination with *Active Plus* and *I Move*. The findings that led to adaptations of the first prototypes are described next. Based on the feedback of interviewees that the addition of instruction videos would be valuable, 3 videos that cover the information in manuals A, B, and C were developed and integrated with the online interventions. Additionally, the majority expected that the concept of setting weekly step goals would be challenging and could motivate them to increase their activity levels. However, some interviewees stated that only setting step goals would not motivate them enough. They preferred to set up a more long-term goal that motivates them to increase their steps, such as a health-related goal. Based on this information, a “motivation-box” was added to the schemes of manuals B and C, where the users can fill in their long-term goals. Lastly, the interviewees indicated that they would like to receive more practical tips on how to increase or maintain the step count in daily life. Therefore, advices regarding this topic were added to *Active Plus* and *I Move*. An overview of the adapted prototype of the intervention including the activity tracker is shown in Figure 3. In this figure, the intervention *I Move* is used as an example. In general, the same structure is used for *Active Plus*. However, *Active Plus* consists only of 2 advices, whereas *I Move* consists of 4 sessions. As a result, *Active Plus* participants receive the activity tracker information about sessions 2 and 3 of *I Move* combined in advice 2.

#### Steps 5 and 6: Pilot Tests, Interviews, and Fine-Tuning Prototypes

Pilot-test participants of the interventions including the activity tracker had a mean score of 77.5 (SD 9.9), which is relatively high on the SUS. Additionally, reported days of use, enjoyment, satisfaction, and motivation scores were the highest among the 3 mobile elements (Table 3). In line with this, it emerged that participants faced little to no technical difficulties. Further, participants reported that the activity tracker is less suitable for people who prefer activities other than walking, since activities such as cycling and swimming need to be specifically started via the workout function of the tracker. In contrast, steps are continuously monitored. Additionally, the step goal schemes are walking oriented as well. Based on these findings, the following adaptations were made to the prototypes including the activity tracker: Information was added to the step goal schemes on how to attach the tracker around the ankle to express the cycling movement in steps, and some missing information regarding installation and use of the tracker was added to the instruction manual. Results regarding EMI and the chatbot are described next.

**Table 3:** Summary results of the evaluation questionnaire, daily testing questionnaire, and compliance with EMI^a^.

Questionnaires			Activity tracker, mean (SD)		EMI, mean (SD)		Chatbot, mean (SD)
**Evaluation questionnaire**							
	SUS^b^ (0-100)	77.5 (9.9)		82.2 (16.8)		52.9 (22.7)	
	Enjoyment (1-10)	8.1 (1.0)		7.4 (1.8)		5.9 (2.1)	
	Satisfaction (1-10)	7.5 (1.3)		7.3 (2.3)		5.1 (2.8)	
**Daily testing questionnaire**							
	Days of use per participant	13.5 (1.1)		11.9 (3.3)		9.0 (5.1)	
	Daily degree of motivation (1-10)	6.7 (2.4)		5.1 (3.0)		5.0 (2.6)	
	Daily enjoyment (1-10)	7.8 (1.4)		5.3 (2.9)		5.0 (2.5)	
Compliance with EMI (%)			N/A^c^		71.8 (27.3)		N/A

^a^EMI: ecological momentary intervention.

^b^SUS: System Usability Scale.

^c^N/A: not applicable.

### Intervention Including EMI

In the next sections, the results of the prototype development phase of the intervention programs including EMI are described.

#### Steps 1 and 2: Literature Search and Development of the First Prototypes

Substantial PA research has been conducted using ecological momentary assessment (EMA). However, studies combining it with EMI, where participants are also provided with a tailored advice after completing each EMA, are surprisingly scarce for PA [48]. EMI programs for smoking cessation [24,25], anxiety [49], and weight loss [50] are more common. Although different types of technology are used to deliver EMI messages, we decided to use short messaging service (SMS) texts, given the expectation that our target population is most familiar with this method. Since text messages are sent via the software of our online interventions, account variables of participants, such as gender and age, gathered during the intervention were used for tailoring within the EMI program.

Although the literature reveals no conclusive recommendations regarding an acceptable frequency for EMI messages, the period over which they are delivered probably plays an important role. A trade-off had to be made between intervention intensity (possibly related to effects) and participant load, since participants accept in general more frequent EMI when the intervention duration is shorter. A protocol where the message frequency decreases as the intervention progresses could be considered as well [51]. Given the relatively long duration of our online interventions of 12 weeks, we decided to follow a decreasing-frequency protocol. For example, in the first week, participants receive 3 text messages per day, whereas in the sixth week, participants receive 4 text messages per week. The time prompts could be user initiated, fixed, or random. Considering the technical capabilities of the software used, we decided to plan the time prompts in advance according to 3 time windows during the day (8:00-11:00 a.m., noon-3:00 p.m., and 5:00-8:00 p.m.). To avoid predictability, text messages are sent at different times within these windows.

To tailor the EMI messages, a short assessment questionnaire precedes each advice moment, which increases the acceptability of the intervention [52]. After clicking the hyperlink provided in the text message, a questionnaire opens, which assesses participants’ current state and context. The questionnaire consists of 6 questions related to the topics current PA level, intention and barriers to be active, energy levels, and stress levels. After filling in this short questionnaire, participants receive a tailored advice related to PA based on the answers directly afterward. In the beginning, participants receive more questionnaires and advices per day, which slowly decreases during the 12 weeks. For this, a relatively large message database is needed. Since the performance objective of the EMI element is to decrease barriers and motivate participants during the day, a literature search regarding the barriers and motivators for adults aged over 50 years to participate in PA was performed. Table 4 shows that the primary barriers differ based on gender, age, and activity level [53,54].

**Table 4:** Primary barriers for adults aged over 50 years to participate in PA^a^.

Barrier	Gender [53]			Age [53]			PA level [54]	
	Men	Women	50-60 years		>60 years	Inactive adults over 50 years		Active adults over 50 years
Lack of motivation	Applicable	N/A^b^	Applicable		N/A	Applicable		Applicable
Perceived abilities and (fear of) pain	N/A	Applicable	N/A		N/A	N/A		N/A
Lack of time	N/A	N/A	Applicable		N/A	N/A		Applicable
Injury	N/A	N/A	N/A		Applicable	N/A		N/A
Poor health	N/A	N/A	N/A		Applicable	N/A		Applicable
Fear of falling	N/A	N/A	N/A		N/A	Applicable		N/A

^a^PA: physical activity.

^b^N/A: not applicable.

Motivators to participate in PA for adults aged over 50 years are health, concerns for the future, enjoyment, PA to regulate stress, increase of social integration, and relational goals [55]. Based on these barriers and motivators, a database of EMI messages was formed. Examples of topics included in the database are benefits of PA, muscle-strengthening exercises, and tips for planning PA. Lastly, an instruction manual was developed on how to use the EMI program, where the same design guidelines were applied as mentioned earlier for the activity tracker.

#### Steps 3 and 4: Semistructured Interviews and Adapting the First Prototypes

**Figure 4:**
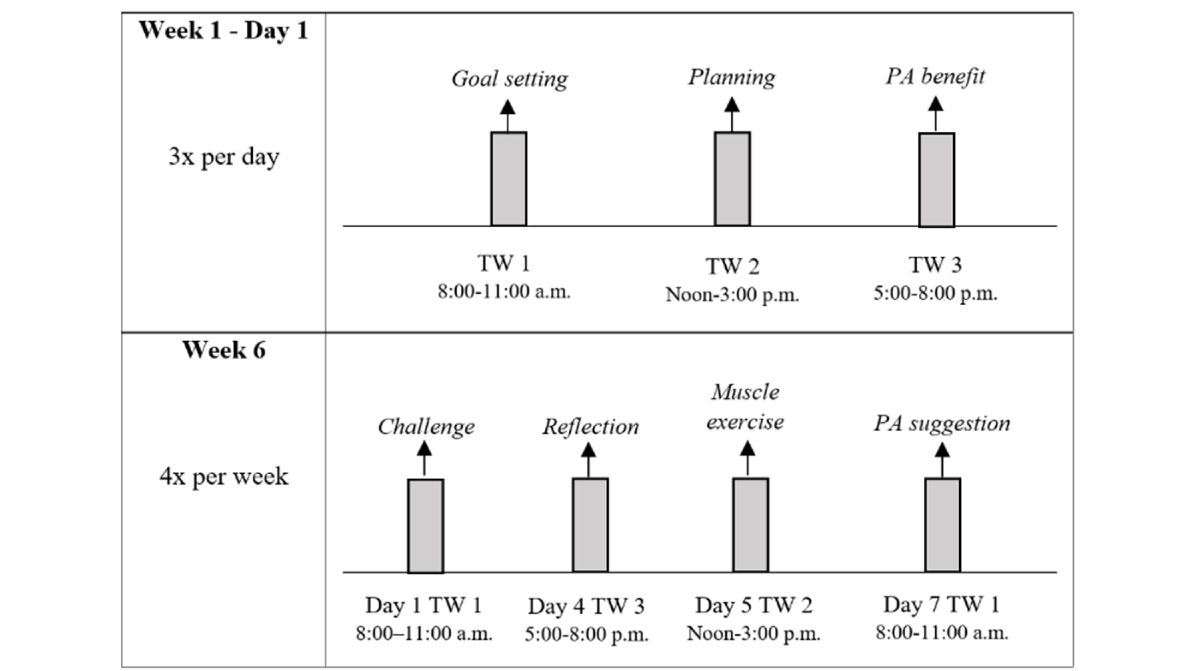
Example of the EMI protocol. EMI: ecological momentary intervention; PA: physical activity; TW: time window.

The themes resulting from the semistructured interviews regarding EMI were (1) barriers to participate in PA, (2) the general principle, (3) technical feasibility, (4) reaching the target population, (5) the EMA questionnaire, (6) intervention messages, (7) frequency, (8) opinion on the testing procedure, (9) name of the EMI program, and (10) combination with *Active Plus* and *I Move*. Based on the interviews, some adaptations were made to the first EMI prototype. First, the EMA questionnaire was fine-tuned since interviewees indicated that they missed some answer options and the definition of the PA concept. Second, during the testing procedure, it was noticed that interviewees faced difficulties while filling in the Likert-scale questions. As most interviewees clicked the number instead of the bar above it, instructions for answering these questions were added. Third, more visual elements, such as images and videos, were added to the text-based EMI messages since interviewees highly appreciated the combination of textual and visual elements. Lastly, interviewees indicated that they missed information regarding the frequency of EMI messages. Therefore, a table with information regarding the frequency of text messages per week was added to the manual. Figure 4 gives an example of the EMI protocol.

#### Steps 5 and 6: Pilot Tests, Interviews, and Fine-Tuning Prototypes

From the pilot test and interviews of the intervention including EMI, it emerged that participants faced some technical issues, which are described in more detail in Textbox 1. This could have influenced the average reported days of use, enjoyment, satisfaction, and motivation scores resulting from the pilot test, which were considerably lower in the EMI group compared to the activity tracker group. In contrast, average SUS scores for EMI were slightly higher compared to the activity tracker (Table 3). Additionally, participants reported that text messages during the second time window were often difficult to answer as a result of work activities. Lastly, participants experienced the advices as stand-alone messages, and they missed the connection with previous received EMI advices. Based on these findings, the following adaptations were made to the prototypes including EMI: The technical issues reported in Textbox 1 were solved, a couple of messages during the second time window were moved to a time when people often have their lunchbreak, and references to previously answered questions and advices were added to the introductory text of new EMA questionnaires to improve the connection between the follow-up advice and the previous advice. More information regarding the EMI pilot test and interview results, together with results from the activity tracker and chatbot, is provided in Table 3 and Textbox 1. Results regarding the chatbot are described next.

**Technical issues participants faced during the interventions.**

**Activity tracker**

- Add specific installation instructions for the activity tracker app for iOS devices.
- Refer to the instruction manual manufacturer for more/other functions of the tracker.
- Visibility/size screen tracker.
- The activity tracker focuses on step count registration. Other activities, such as cycling, swimming, and household activities, are more difficult to measure and do not contribute to the achievement of the step goal.

**Ecological momentary intervention (EMI)**

- Some technical issues when switching between pages.
- One participant reported that they received only 1 text message per day, although they were sent from our system.
- Possibility to review all previous advices.
- More personalized/tailored messages needed.
- More interaction with the baseline questionnaire (T_0_) and tailoring messages for EMI.
- Personalized timing messages, considering factors such as work.
- Not all advices sort with COVID-19 restrictions at the time of the pilot test (eg, curfew, closure of gyms and swimming pools).

**Chatbot**

- The installation and use of 2 apps (step count + messages) is seen as confusing. One combined app is preferred.
- Participants often already use similar apps, which they find more sophisticated.
- Focus only on walking and not on other activities.
- The step counter needs to be reactivated regularly to work properly.
- The step counter is not usable on older types of smartphones.
- Participants were regularly automatically logged out of the step count app.
- Technical difficulties in exchanging baseline (T_0_) variables with the chatbot database.

### Intervention Including the Chatbot

In the subsequent sections, the results of the prototype development phase of the intervention including the chatbot are described.

#### Step 2: Development of the First Prototypes

An already existing just-in-time walking coach chatbot [27] was adapted according to the needs of this project. This chatbot was originally developed for the Supreme Nudge project [31] and provides personalized walking messages on specific moments of choice via the Telegram chat app. The aim of these messages is to increase (the motivation for) walking during the day. Tailoring of the BCT message is performed by consideration of baseline data on personal BCT preferences, added to the baseline questionnaires of *Active Plus* and *I Move*. Additionally, a secure and GDPR-compliant data structure was built to enable ongoing exchange of data from the intervention software to the chatbot database.

Furthermore, contextual variables assessed via users’ smartphones, such as the step count measured via a separate app, were used for message tailoring. The chatbot used during the Supreme Nudge project possessed a function based on the geographical location of green spaces, where manual mapping of the environment based on coordinates was needed. As that study was conducted in the geographical area of Amsterdam, manual mapping was feasible. However, recruitment during this study took place on a national level, which impeded the use of the green space function. The green space function of the chatbot was therefore deactivated during this study.

Since previous research shows that good weather conditions are a facilitator for being physically active outside, whereas bad weather conditions are a hindering factor for being physically active outside [56,57], a weather function was added to the chatbot. Especially in the Netherlands, considering its unpredictable and changing weather conditions, message tailoring based on weather is valuable. Weather is also an important factor for walking behavior for those with migraine, rheumatism, or hay fever, which is common in our target population [58]. For the weather function, a data structure was built where weather ratings can be automatically extracted from a Dutch weather forecast website based on the residence of a participant. The ratings are calculated automatically by this website, based on rules composed by meteorologists, considering various factors, such as rain, wind, and temperature. The residence is questioned in the baseline questionnaire and is sent to the chatbot database via the developed ongoing data structure. The weather ratings are used to tailor the messages according to the current weather conditions. Additionally, weather ratings related to the limitations migraine, rheumatism, and hay fever are extracted. Complaints related to these limitations are, namely, influenced by weather conditions. For example, migraine attacks can be provoked by an acute drop in air pressure prior to an approaching thunderstorm. All participants receive tailored messages considering the general weather ratings of the day in their reported residence. Additionally, those indicating in the baseline questionnaire that they have 1 or more of the mentioned limitations receive specific messages based on the limitation-related weather ratings. The weather function is used as an additional boost for the intervention, since it was introduced after the second advice/session (Multimedia Appendix 1).

Data exchange is also possible from the step count app database to the online interventions. Within the advices of *Active Plus* and the sessions of *I Move*, this information is used to provide participants with tailored feedback on their step count. These advisory texts resemble the activity tracker step count texts, as presented in Multimedia Appendix 6. Lastly, a manual was developed with instructions on how to install and use the chatbot, where the same design guidelines were applied as mentioned earlier for the activity tracker and EMI.

#### Steps 3 and 4: Semistructured Interviews and Adapting the First Prototypes

**Figure 5:**
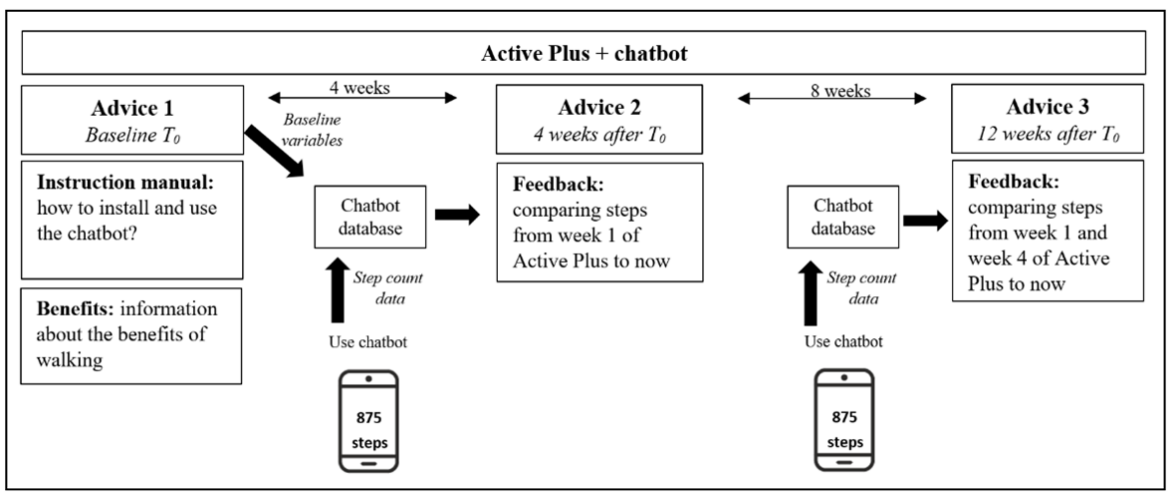
Overview of the online intervention Active Plus including the chatbot.

The themes resulting from the semistructured interviews regarding the chatbot were (1) general principle, (2) technical feasibility, (3) reaching the target population, (4) messages, (5) opinion on the testing procedure, (6) instruction manual, and (7) combination with *Active Plus* and *I Move*. Based on the interviews, the chatbot instruction manual was fine-tuned since interviewees indicated that they missed some interim instruction steps for installation. Further, interviewees indicated that they preferred to receive some background information about the benefits of walking and use of the chatbot within the first advice of *Active Plus* and the first session of *I Move*. As a result, information regarding these benefits was added to the online interventions. In general, interviewees were concerned that the chatbot would not measure their steps correctly, since they do not always carry their phones with them. This could influence the tailoring with regard to the step count variable and therewith lead to the selection of inappropriate messages. To respond to this, a disclaimer was added to the chatbot instructions describing this problem. A schematic overview of the intervention *Active Plus* including the chatbot is given in Figure 5.

#### Steps 5 and 6: Pilot Tests, Interviews, and Fine-Tuning Prototypes

From the pilot test and interviews of the intervention including the chatbot, it appeared that participants faced several technical problems. The evaluation and daily-testing questionnaire scores shown in Table 3 reflect this, since the chatbot group scored the lowest among the 3 mobile elements. First, participants reported that they were logged out automatically from the step count app and needed to reactivate it regularly. Second, it appeared that the exchange of variables in both directions between the intervention software and the chatbot database took place too infrequently. Based on these findings, the following adaptations were made to the prototypes including the chatbot: The connection between the database and the step count app was strengthened and the data structures between the intervention software and the chatbot database were improved to effectuate more regular exchange of variables in both directions. As a result, it is expected that the technical problems are solved and the usability of the chatbot has improved. An overview of the pilot test and interview results of the 3 mobile elements is provided in Table 3 and Textbox 1.

## Discussion

### Principal Findings

During this prototype study, literature searches were executed, interviews were held, and pilot tests were performed. The aim was to enhance 2 existing PA-stimulating computer-based interventions with 3 mobile elements (an activity tracker, an EMI program, or a chatbot) and test the prototypes on usability and appreciation within the target population of adults aged over 50 years. Although the pilot-test results showed that all prototypes scored moderate to good on usability and appreciation, the scores differed between the mobile elements.

The developed activity tracker intervention was the most usable and highest-appreciated prototype. Additionally, no major technical difficulties were faced during the pilot test of this prototype. In contrast, the EMI and chatbot participants faced several technical issues while pilot-testing the prototypes. The lower, but still moderate, usability and appreciation rates of the EMI and chatbot interventions compared to the activity tracker could be explained by these issues. Based on the information provided by participants, the technical difficulties faced could be solved, which is expected to improve the usability and appreciation rates of the EMI and chatbot prototypes.

### Strengths

The design of a systematic approach [28] prior to starting the prototype development is considered a strength of our study since it contributed to the preservation of the already proven effectiveness of the original computer-based *Active Plus* [10] and *I Move* [11] interventions. Additionally, the intention of co-designing the prototypes with our target population of adults aged 50 years and over is a strength since it has been shown to be crucial for successful eHealth design [59,60].

### Limitations

However, a limitation in line with this is that practical execution of the target population involvement was impeded in this study. The prototype development phase took place in a period when society was shut down as a result of the COVID-19 pandemic. Interview results were affected by this since it was difficult to conduct face-to-face interviews with our target population. As a result, our interview samples were smaller than previously intended, and fewer older adults aged over 70 years with low education and low digital skills were reached. The same applies to the pilot-test sample since it was difficult to reach populations with low digital (health) skills. Although this situation was not ideal, we tried to respond to this as adequately as possible. First, we applied the knowledge gathered in our previous eHealth studies conducted within vulnerable populations to the prototype development in this study [14,61,62]. Second, more emphasis was put on design guidelines for those with lower literacy during the literature searches in the first step of the design process. Lastly, interviewees were asked to put themselves in the position of older persons, those with low education, or persons with lower digital skills whom they knew from their environment (eg, someone in their family, neighbor, or friend), in addition to their own opinions and experiences regarding the mobile elements. This combined approach led to valuable design guidelines for vulnerable populations, which contributed to the prototype development process. However, for future research, it is recommended that the prototypes are tested on a larger sample of those with low education and low digital (health) skills. It has namely been shown that design preferences with regard to eHealth interventions differ for these subpopulations compared to those with higher education and with higher digital skills, which is often overlooked [63].

The limited time available to execute the separate steps of the design approach is considered another limitation of the study. An example is that we were therefore forced to perform broad literature searches instead of systematic literature searches in step 1. As a result, we cannot be completely sure that we included all relevant studies for the prototype development. Additionally, developments regarding mHealth technologies and the commercial activity tracker market are quick. New insights from 2021 and later were not included in this study.

### Conclusion

Despite the mentioned limitations, it can still be concluded that the developed prototypes were sufficiently usable and appreciated by the target population of adults aged 50 years and over, according to the objective of this study. Based on the results, it can be concluded that the integration of an activity tracker with a computer-based PA intervention is the most promising option among the 3 added and tested mobile elements. The earlier developed systematic approach proved useful for the purposes of this study since the prototypes with the added mobile elements scored moderate to high on usability and appreciation. Other eHealth and mHealth developers are therefore recommended to use the approach as a guideline during their own prototype development process, especially in situations in which already existing interventions are extended or renewed. In the next phase, effects of the interventions can be evaluated through a randomized controlled trial, known as the effect evaluation phase of our systematic design approach.

## Appendix

Multimedia Appendix 1 Overview of the structure of the computer-based interventions Active Plus and I Move.

Multimedia Appendix 2 Insight into the computer-based interventions Active Plus and I Move.

Multimedia Appendix 3 Semistructured interview guide for the activity tracker.

Multimedia Appendix 4 Semistructured interview guide activity tracker example questions.

Multimedia Appendix 5 Market study of activity trackers.

Multimedia Appendix 6 Example advisory texts step count AT and CB Active Plus and I Move.

## Abbreviations

BCT: behavior change technique
EMA: ecological momentary assessment
EMI: ecological momentary intervention
GDPR: General Data Protection Regulation
mHealth: mobile health
MVPA: moderate-to-vigorous physical activity
PA: physical activity
SUS: System Usability Scale
TAM: technology acceptance model
UTAUT: unified theory of acceptance and use of technology

## Data Availability

The data sets from which the results of this study were derived can be obtained from the corresponding author upon reasonable request.
