# Supplementary material for: Adding Mobile Elements to Online Physical Activity Interventions for Adults Aged Over 50 Years: Prototype Development Study
Source: JMIR Form Res. 2023 Jan 25;7:e42394. doi: 10.2196/42394 (PMC9909523; doi:10.2196/42394)
Supplement: Multimedia Appendix 1 [file formative_v7i1e42394_app1.docx]

**Appendix 1 – Overview structure computer-based elements Active Plus and I Move**

**
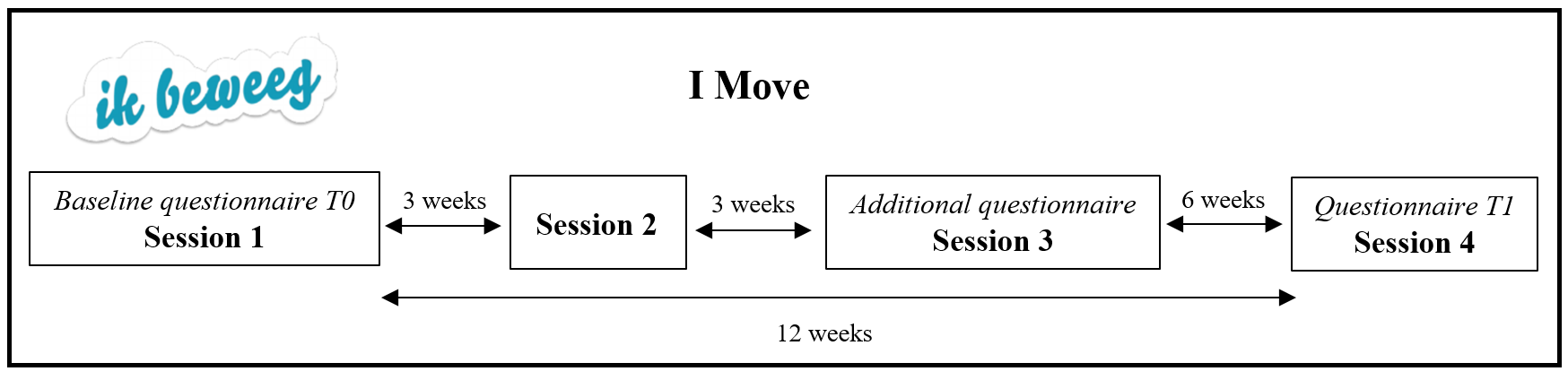

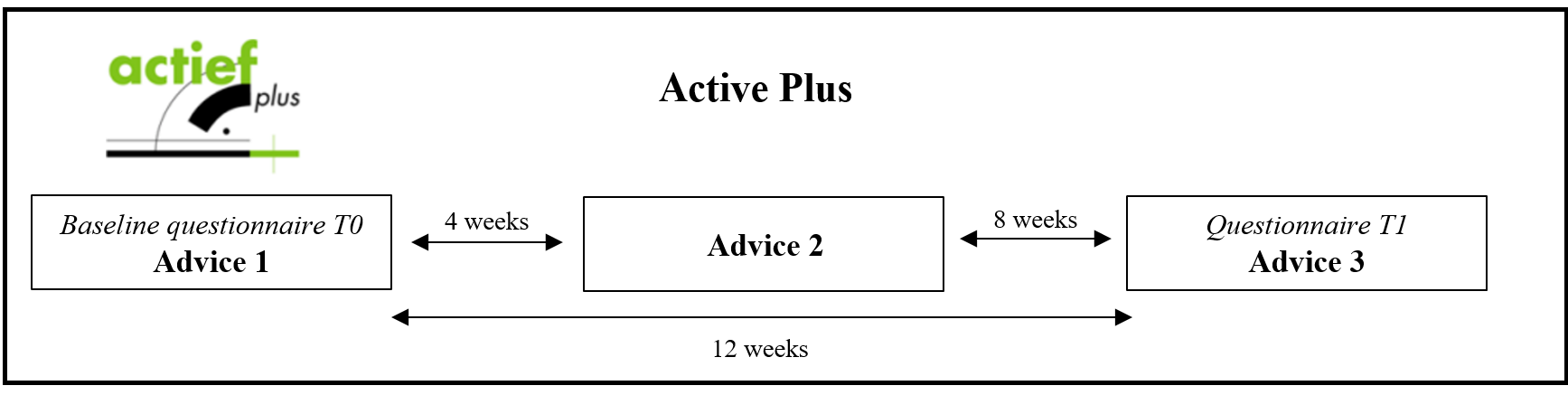
**

**Figure 6. Schematic overview computer-based elements Active Plus**

**Figure 7. Schematic overview computer-based elements I Move**
