# Supplementary material for: Adding Mobile Elements to Online Physical Activity Interventions for Adults Aged Over 50 Years: Prototype Development Study
Source: JMIR Form Res. 2023 Jan 25;7:e42394. doi: 10.2196/42394 (PMC9909523; doi:10.2196/42394)
Supplement: Multimedia Appendix 2 [file formative_v7i1e42394_app2.docx]

**
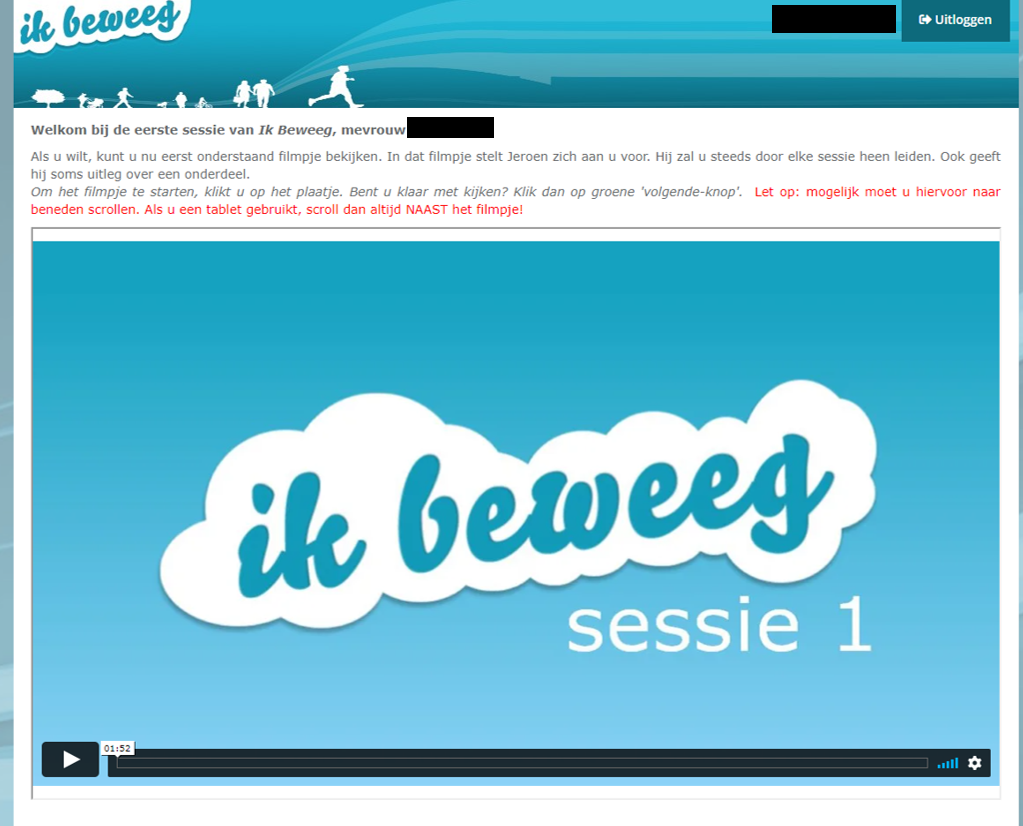

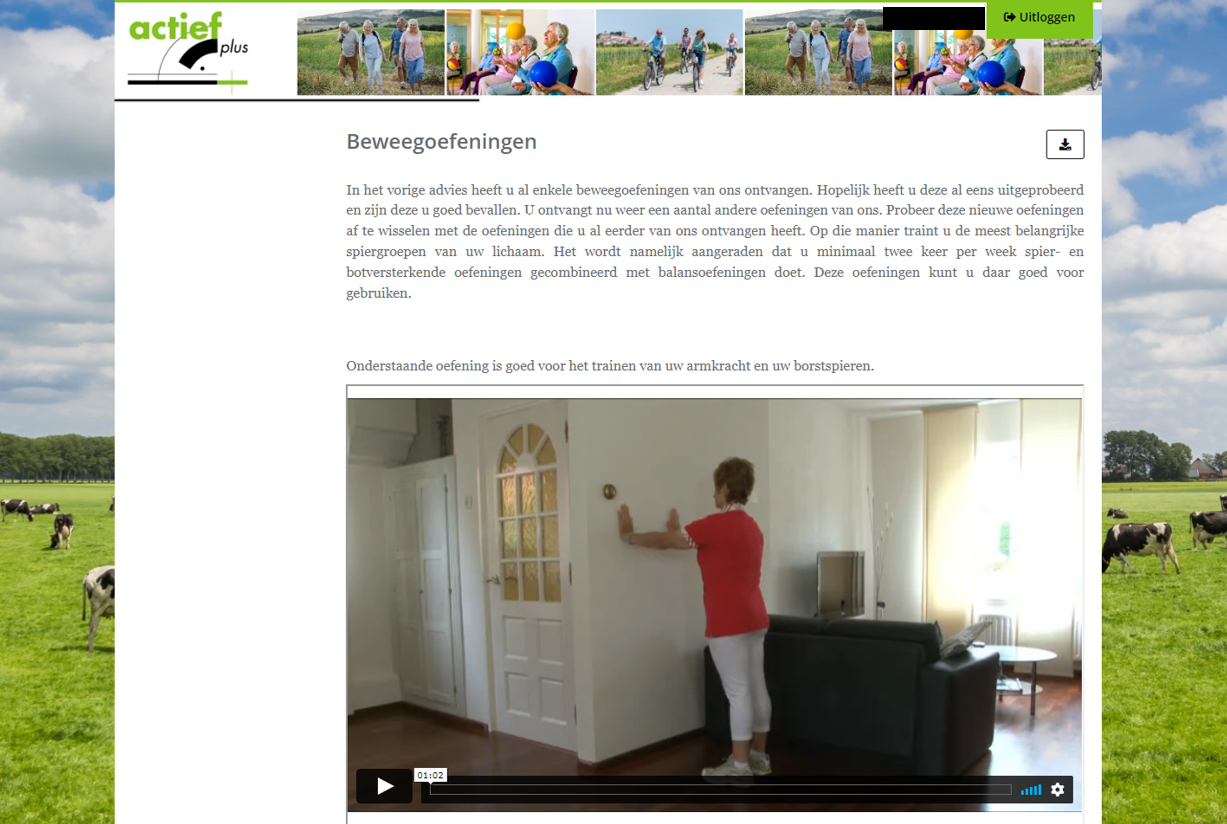
Appendix 2 – Insight into computer-based interventions Active Plus and I Move**

**Figure 9. Example advisory text I Move**

In this example, the introductory page of the first session of I Move is shown. Instructions regarding the session and program are given and virtual coach Jeroen introduces himself via a video.

**Figure 8. Example advisory text Active Plus**

In this example, the participants receives tailored bone- and muscle strengthening exercises. The exercise is presented with both textual and video instructions.
