# Supplementary material for: Adding Mobile Elements to Online Physical Activity Interventions for Adults Aged Over 50 Years: Prototype Development Study
Source: JMIR Form Res. 2023 Jan 25;7:e42394. doi: 10.2196/42394 (PMC9909523; doi:10.2196/42394)
Supplement: Multimedia Appendix 3 [file formative_v7i1e42394_app3.docx]

**Appendix 3 – Semi-structured interview guide activity tracker**

|  | **Face-to-face** | **Digitally** |
| --- | --- | --- |
| 1. **Introduction Active Plus / I Move** | PowerPoint on large screen (to keep a distance of 1,5 m) | PowerPoint and instruction manuals were mailed just before the start of the interview to the participant + screen sharing. Activity tracker was sent via post. |
| 1. **Explanation additional element activity tracker** |  |  |
| - *Experiences activity tracker* - *Opinion with respect to use* |  |  |
| 1. **First tests Mi Fit Band without manuals 🡪 think-aloud** | Give Mi Fit band to participant. First general testing of Mi Fit Band + discuss screenshots PowerPoint | First general testing of Mi Fit Band + discuss screenshots Powerpoint |
| *- First impressions?*  *- Improvements?* |  |  |
| 1. **Installation and testing Mi-Fit App using instruction manual A** | Ask participant to install and test application on smartphone using instruction manual A | Ask participant to install and test application on smartphone using instruction manual A |
| - *Experiences regarding use (health) applications* - *Instructions on installation* - *First impression Mi Fit application: improvements, additional value* - *Opinion with respect to use* |  |  |
| 1. **Show, explain, and testing step-goal manuals B and C** | Paper-based versions manual B and C | Online versions manual B and C + screen sharing |
| - *Step-goal concept* - *Usability manuals* - *Language use* - *Lay-out* |  |  |
| 1. **Explanation integrating activity tracker with existing Active Plus and I Move intervention** |  |  |
| - *Additional value* |  |  |
| 1. **Show examples integration activity tracker with Active Plus and I Move** | Screenshots PowerPoint Active Plus and I Move advices related to activity tracker | Screenshots PowerPoint Active Plus and I Move advices related to activity tracker |
| - *Opinion?* - *Preferences?* - *Visual / textual / combination?* |  |  |
