# Supplementary material for: Adding Mobile Elements to Online Physical Activity Interventions for Adults Aged Over 50 Years: Prototype Development Study
Source: JMIR Form Res. 2023 Jan 25;7:e42394. doi: 10.2196/42394 (PMC9909523; doi:10.2196/42394)
Supplement: Multimedia Appendix 4 [file formative_v7i1e42394_app4.docx]

**Appendix 4 – Semi-structured interview guide activity tracker example questions**

| *Experiences activity tracker* |
| --- |
| - Have you ever heard of an activity tracker? - Do you know what an activity tracker is? - Have you ever used and activity tracker? If yes, experiences? If no, why not? - Would you use it (again)? Why yes/no? |
| *Opinion with respect to use (after explanation in case needed)* |
| - Do you think an activity tracker could help you to be more physically active? Why yes/no? - Which barriers/obstacles do you see in using an activity tracker? - Do you think you would like to use an activity tracker for a longer period of time (3-4 months)? - Which functions for an activity tracker are important in your opinion (give examples to participant in case needed)? |
| *Mi Fit Band think-aloud testing procedure* |
| - Positives? - Negatives? - Easiness of use? - Improvements? - Instructions needed for proper use? - How do you think other people your age would experience this tracker? - How do you think people older than you would experience this tracker? - How do you think people with low digital skills would experience this tracker? - Would you spend money for this activity tracker yourself? How much approximately? |
| *Mi Fit application* |
| - What are your experiences regarding the use of (health) applications? - Do you think an application is an useful addition for an activity tracker? - What is your first opinion regarding this application? - Would you use this application (in addition to the information given by the tracker)? Why yes/no? - What do you think of the instructions in the manual to install the application on your smartphone? |
| *Instruction manual A – Preparing for use and using the activity tracker* |
| - Do you think you will manage to get the Mi Fit application on your smartphone with these instructions? Why yes/no? In case of no, what do you need to be able to install the application successfully? - Do you think these instructions will help you to get the tracker ready for use? - How do you think other people your age experience the instructions in the manual? - How do you think people older than you experience the instructions in the manual? - How do you think people with low digital skills experience the instructions in the manual? - How do you think low literate experience the instructions in the manual? - Which improvements can be made to the manual? |
| *Instruction manuals B + C – Step goals* |
| - Do you think setting step goals could help you to be (more) physically active? - Would you choose to set step goals with the application or with paper-based schemes? Why? - Would you use the schemes? Why yes/no? - Is it directly clear how the schemes need to be filled in or is the additional provided information needed? - Improvements for the manuals and/or schemes? (textual, explanation, lay-out etc.) - Is it clear which manual you need to use in which situation (manual B of C)? |
| *Intervention Active Plus/I Move + activity tracker* |
| - Would the activity tracker be an addition to the existing computer-based Active Plus/I Move? - Do you think you would be more physically active if the activity tracker is part of Active Plus/I Move (as compared to only the computer-based advices) - Do you think you would be more motivated to be physically active if the activity tracker is part of Active Plus/I Move (as compared to only the computer-based advices) - How do you think the activity tracker can best be combined with the existing advices/sessions of Active Plus/I Move - What do you think of this example (interviewer shows examples of integration activity tracker with advices Active Plus / sessions I Move)? |
