# Supplementary material for: Adding Mobile Elements to Online Physical Activity Interventions for Adults Aged Over 50 Years: Prototype Development Study
Source: JMIR Form Res. 2023 Jan 25;7:e42394. doi: 10.2196/42394 (PMC9909523; doi:10.2196/42394)
Supplement: Multimedia Appendix 5 [file formative_v7i1e42394_app5.docx]

**Appendix 5 – Market study activity trackers**

**Table 6. Summary market study activity trackers**

| **Tracker** | **Costs^1^** | **Accuracy** | **Step count** | **Stair count** | **Heart rate** | **Calorie meter** | **Screen size (inch)** | **Screen resolution** | **Display type^2^** | **Touch screen** | **Battery life (days)** | **Charge time (min)** | **GPS** | **Social component** |
| --- | --- | --- | --- | --- | --- | --- | --- | --- | --- | --- | --- | --- | --- | --- |
| Xiaomi Mi Band 2 OLED | € 26,90 | Yes | Yes | No | Yes | No | 0.42 | 72x40 | OLED | No, button | 21 | 150 | No | Yes |
| Huawei Band 3e | € 32,00 | Unknown | Yes | Yes | No | Yes | 0.50 | 480x800 | PMOLED color screen | Yes | 12 | Unknown | Yes | Yes |
| Samsung Galaxy fit e | € 38,95 | Unknown | Yes | Yes | Yes | Yes | 0.75 | 128x64 | AMOLED | Yes | 7 | Unknown | No | Yes |
| Xiaomi Mi Smart Band 4 | € 39,00 | Yes | Yes | No | Yes | Yes | 0.95 | 120x240 | Color RGB AMOLED | Yes | 20 | ≤ 120 | No | Yes |
| Fitbit Inspire | € 68,19 | Yes | Yes | No | No | Yes | 1.4 | 126x36 | Greyscale OLED | Yes | 5 | 60-120 | No | Yes |
| Huawei Band 3 Pro | € 75,00 | Unknown | Yes | Yes | Yes | Yes | 0.95 | 120x40 | Color AMOLED | Yes | 14 | 100 | Yes | Yes |
| Fitbit Charge 3 | € 99,00 | Yes | Yes | Yes | Yes | Yes | 1.57 | 100x150 | Greyscale OLED | Yes | 7 | 60-120 | No | Yes |
| Garmin Vivosport | € 105,00 | Unknown | Yes | Yes | Yes | Yes | 0.38 | 72x144 | Transflective MIP | Yes | 7 | 240 | Yes | Yes |

^1^ January 2020, the Netherlands

^2^ AMOLED = active-matrix organic light-emitting diode ; MIP = memory-in-pixels ; OLED = organic light-emitting diode ; PMOLED = passive-matrix organic light-emitting diode
