# Supplementary material for: Adding Mobile Elements to Online Physical Activity Interventions for Adults Aged Over 50 Years: Prototype Development Study
Source: JMIR Form Res. 2023 Jan 25;7:e42394. doi: 10.2196/42394 (PMC9909523; doi:10.2196/42394)
Supplement: Multimedia Appendix 6 [file formative_v7i1e42394_app6.docx]

**Appendix 6 – Example advisory texts step count AT and CB Active Plus and I Move
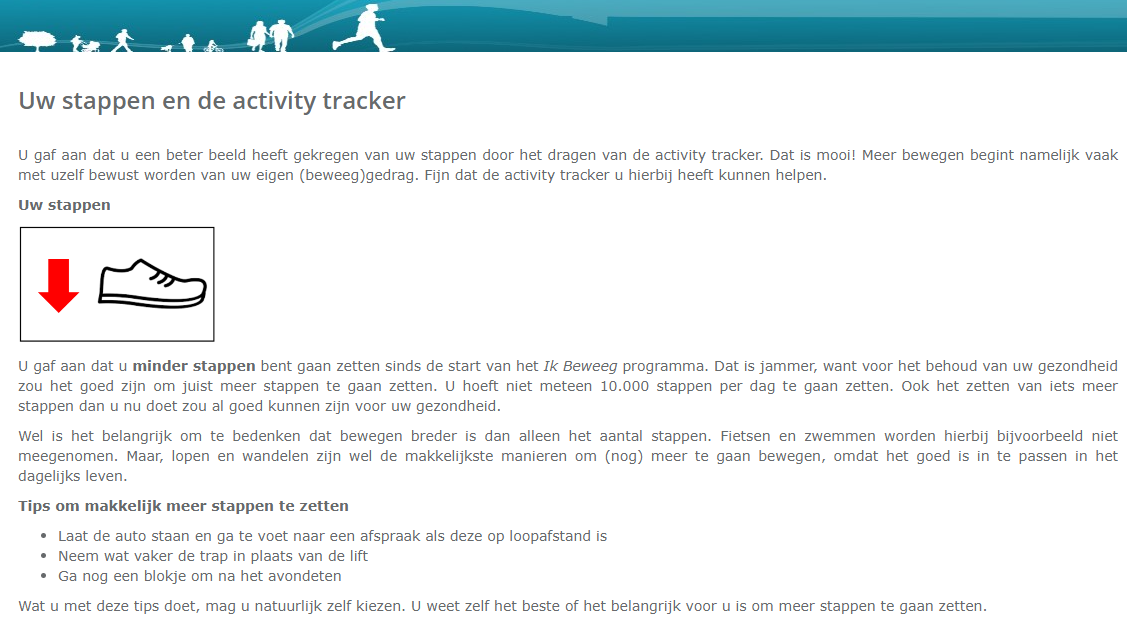
**

Practical tips for increasing steps in daily life

Visual presentation of step behavior in time. In this example, the participants’ steps decreased from session 1 to session 2 of I Move.

Insight into step behavior / awareness

**Figure 10. Example advisory text in I Move regarding step count activity tracker**

Comparable advisory texts regarding step count are used for the chatbot within Active Plus and I Move.
